# Integrated Proteomic and Lipidomic Profiling of Aqueous Humor Reveals Inflammatory Signatures in Retinitis Pigmentosa

**DOI:** 10.3390/biomedicines14061259

**Published:** 2026-05-31

**Authors:** Leonardo Colombo, Anna Caretti, Salvatore Martella, Andrea Corona, Linda Montavoci, Michele Dei Cas, Jacopo Baldesi, Roberta Rissotto, Chiara Quisisana, Alessandro Autelitano, Filippo Martinelli Boneschi, Luca Rossetti

**Affiliations:** 1 Eye Clinic, ASST Santi Paolo e Carlo Hospital, Università degli Studi di Milano, 20142 Milan, Italy; leonardo.colombo@asst-santipaolocarlo.it (L.C.); jacopo.baldesi@unimi.it (J.B.); roberta.rissotto@gmail.com (R.R.); chiara.quisisana@gmail.com (C.Q.); alessandro.autelitano@unimi.it (A.A.); luca.rossetti@unimi.it (L.R.); 2 Biochemistry and Molecular Biology Laboratory, Department of Health Sciences, Università degli Studi di Milano, 20142 Milan, Italy; anna.caretti@unimi.it (A.C.); linda.montavoci@unimi.it (L.M.); michele.deicas@unimi.it (M.D.C.); 3 Laboratory of Precision Medicine of Neurological Diseases, Aldo Ravelli Center for Neurotechnology and Experimental Brain Therapeutics, Department of Health Sciences, Università degli Studi di Milano, 20142 Milan, Italy; andrea.corona@unimi.it (A.C.); filippo.martinelli@unimi.it (F.M.B.)

**Keywords:** retinitis pigmentosa, bioactive lipids, inflammatory protein, aqueous humor

## Abstract

**Background/Objectives:** Retinitis pigmentosa (RP) is characterized by progressive degeneration of photoreceptors, with increasing evidence supporting the involvement of inflammation in disease progression. Aqueous humor (AH) reflects the intraocular microenvironment and represents an accessible source for biochemical analysis. This study aimed to characterize the profile of the main inflammatory proteins and bioactive lipids in the AH of RP patients and to compare it with healthy subjects. **Methods:** The AH was analyzed for cytokines using multiplex immunoassays and for lipid species using liquid chromatography–mass spectrometry. The concentrations of the analyzed molecules were compared between RP patients and the control group and then correlated with age and ellipsoid zone (EZ) width in RP patients. **Results:** A total of 26 RP patients and 13 controls were recruited. Significantly elevated levels of the pro-inflammatory IL-6 and a significant decrease in vascular endothelial growth factor (VEGF) were found in RP patients compared to controls. In RP patients, lipidomic analysis demonstrated significant increases in medium- and long-chain sphingomyelins (SMs) and very-long-chain unsaturated phosphatidylcholines (PCs). Higher levels of Cer 16:0, PC 32:0, and PC 34:0 were significantly associated with greater EZ preservation in RP patients. Additionally, in RP patients, VEGF and GM-CSF levels increased significantly with age, while IL-8 showed a non-significant decreasing trend. **Conclusions:** By integrating proteomic and, for the first time, lipidomic analyses of AH, we identified significant alterations in pro-inflammatory cytokines and bioactive lipid species in RP patients compared to controls, further highlighting a link between inflammatory activity, patient age, and disease stage. These preliminary findings need further validation in larger longitudinal cohorts to confirm the clinical utility of these bioactive mediators as potential disease biomarkers.

## 1. Introduction

Retinitis pigmentosa (RP) refers to a group of clinical conditions characterized by the progressive degeneration of photoreceptors in a rod-cone pattern, caused by genetic variants in over one hundred genes [1,2]. The onset of the disease is triggered by the presence of one or more genetic variants, leading to rod degeneration. However, other factors contribute to disease progression, including oxidative stress, inflammation, and the reduced release of trophic factors, which are considered major mechanisms of damage, particularly affecting the cones [3,4]. In recent years, the role of inflammation in RP has been explored by several research groups in both animal models and humans. In animal models, it has been demonstrated that microglial activation and upregulation of inflammatory genes characterize the photoreceptor degeneration phase [3,5,6]. Furthermore, targeting inflammation may provide an effective neuroprotective approach, potentially prolonging cone survival [7,8].

One commonly used method to assess the biochemical changes occurring in the eye is the analysis of ocular fluids [9], particularly aqueous humor (AH), since it can be obtained via minimally invasive procedures. In humans, Yoshida et al. postulated that sustained chronic inflammation may underlie RP pathogenesis by evaluating the presence of inflammatory cells in the anterior vitreous through slit-lamp biomicroscopy and analyzing cytokines in both the aqueous and vitreous fluids of RP patients [10]. Okita et al. found that peripheral markers of inflammation could be associated with visual function in RP patients [11]. Moreover, inflammation has been linked to RP progression and complications, such as post-cataract surgery anterior capsular contraction or epiretinal membrane (ERM) formation [4,12,13,14,15].

AH analysis is increasingly important for understanding the etiopathogenic mechanisms and evaluating responses to therapies, particularly in acquired diseases such as age-related macular degeneration, diabetic retinopathy (DR), glaucoma, and uveitis [16,17,18,19]. Furthermore, Wolf et al. demonstrated that almost 90% of vitreous proteins could also be detected in the AH, reinforcing the usefulness of this approach for deeper insights into pathophysiology and for identifying potential markers of retinal diseases [20].

While protein composition analysis of AH is well-established, the profile and the role of lipids in ocular fluids remain insufficiently explored. As it concerns the role of lipids in inflammation, an increase in pathological vitreous proinflammatory eicosanoids and both bioactive sphingolipids and lysophospholipids potentially implicated in inflammatory pathway has been described [21,22,23]. More evidence regards non-ocular pathologies. Dei Cas et al. reviewed the pro- and anti-inflammatory roles of lipids in systemic autoimmune diseases (e.g., rheumatoid arthritis (RA), systemic lupus erythematosus, type 1 diabetes), highlighting their crucial bioactive role in inflammatory processes by regulating hypervascular reactivity, pain, leukocyte trafficking, and clearance. A shift from an acute to a chronic disease state may be driven by the accumulation of inflammatory lipids [24].

Although there are studies on pro-inflammatory protein mediators (cytokines and chemokines) in RP, the role of pro-inflammatory bioactive lipids remains poorly investigated.

The aim of this study is to characterize the profile of the main inflammatory proteins and bioactive lipids in the AH of RP patients and to compare it with healthy subjects. We also investigated whether protein and lipid mediators correlated with patient age and disease severity and whether these markers could serve as potential biomarkers and therapeutic targets.

## 2. Materials and Methods

### 2.1. Study Population

In this prospective, parallel-group trial, RP patients and healthy controls, all with a clinical indication for cataract surgery, were recruited at the ASST Santi Paolo and Carlo Hospital Eye Clinic, a member of the European Reference Network for Eye Diseases (ERN-EYE). RP diagnosis was based on typical fundoscopy findings, characteristic full field electroretinographic patterns, visual field constriction, and genetic analysis when available.

Exclusion criteria included previous eye surgery or procedures within the past 12 months, glaucoma or any other chronic eye disease, the use of ocular topical drugs (except artificial tears and lubricating eye drops), a history of uveitis or other inflammatory eye diseases (e.g., DR, retinal vascular diseases), and patients using systemic anti-inflammatory drugs or affected by diabetes mellitus, systemic inflammatory diseases, or conditions that could involve the eyes. Patients lacking the psychological and physical capacity to understand the study’s aim or provide informed consent were also excluded.

At the baseline visit, a complete medical and ocular history was obtained to confirm eligibility. Visual acuity, anterior segment and dilated fundus examinations, intraocular pressure (IOP), and spectral domain optical coherence tomography (OCT) were performed before and one month after surgery.

The primary endpoint was the inter-group difference in the concentration of sphingolipid/lipid mediators, cytokines, chemokines, and growth factors in the AH of RP patients and control subjects. Secondary outcomes included the correlation between the concentrations of the different molecules analyzed with patient age and disease stage, as assessed by ellipsoid zone (EZ) band width in OCT.

All participants signed informed consent forms approved by the Ethics Committee before specimen collection. The privacy rights of human subjects were fully respected. All procedures were conducted in accordance with the World Medical Association’s Code of Ethics (Declaration of Helsinki). The study protocol was approved by the Ethical Committee of Milan Area 1 (protocol number 0051879/2021).

### 2.2. OCT Scans Analysis

OCT scans were performed with dilated pupils (using 1% tropicamide eye drops). The EZ width was measured as previously described [25]. Briefly, using SD-OCT and Heidelberg Eye Explorer (HEYEX) software V7.0.4 (Heidelberg Engineering, Heidelberg, Germany), single-line scans crossing the fovea horizontally were analyzed. EZ width was manually measured by two experienced OCT readers using the caliper tool in the HEYEX software, and the values obtained by the two physicians were averaged.

### 2.3. Aqueous Humor Collection

Prior to cataract surgery, approximately 0.05–0.1 mL of undiluted AH was drawn from the anterior chamber using a 30-gauge needle attached to a 1 mL syringe. The AH was immediately stored at −80 °C. To prevent variations in molecule concentrations, patients were prohibited from using any topical drugs (other than antibiotic drops) within 36 h before surgery. Oxybuprocaine hydrochloride (Novesina^®^, Laboratoires Théa, Clermont-Ferrand, France) followed by povidone-iodine (Oftasteril^®^, Alfa Intes Industria Terapeutica Splendore S.r.l., Casoria, Italy) were allowed within 10 min before surgery. Mydrane^®^ (phenylephrine [0.31%], tropicamide [0.02%], and lidocaine [1%]) (Laboratoires Théa, Clermont-Ferrand, France) were administered after AH sample collection to avoid potential interference with the AH composition.

### 2.4. Biochemical Analysis

#### 2.4.1. Cytokine Analysis by ELISA

AH samples from RP patients and healthy controls were analyzed using biomarker multiplex immunoassays on the Luminex^®^ Platform (Thermo Fisher Scientific, Waltham, MA, USA) as previously described [26]. IL-6, IL-8, IL-10, TNF-α, VEGF, and GM-CSF were quantified in duplicate. Standard quality assessment has been run after the conclusion of the assigned work. Luminex Performance Verification kit F3D-PVER-K25 and Luminex Performance Verification kit F3D-PVER-K25 have been used for the QC (Quality Control) Instrument Test. Data obtained have been checked and the following QC parameters obtained: (1) values of the standard curve are compared with the values provided by the manufacturer of the kits, and did not exceed a CV of 15%; (2) all the above parameters have been applied to at least 90% of the standard curve values; (3) the assay has passed the standard QC.

#### 2.4.2. Lipid Determination by LC-MS/MS

The lipidomic profile of key lipid classes—including ceramides (Cer), lysophosphatidylcholines (LPCs), phosphatidylcholines (PCs), and sphingomyelins (SMs)—was assessed in AH samples from RP patients and controls.

Lipid analysis was performed as previously described [26,27,28]. Briefly, 70 µL of sample were used for sphingolipid analysis and 30 µL for phospholipid analysis. Samples were extracted with cold methanol/chloroform (2:1, *v*/*v*). For sphingolipids, alkaline methanolysis was performed (75 µL KOH 1 M, 2 h at 38 °C). The extract was analyzed using LC-MS/MS on a Dionex 3000 UltiMate system (Thermo Fisher Scientific) coupled to an AB Sciex 3200 QTRAP tandem mass spectrometer (AB Sciex, Concord, ON, Canada) with electrospray ionization (ESI+). Lipid species were separated on an Acquity BEH C8 column using a gradient of mobile phases (A) water + 0.2% formic acid + 2 mM ammonium formate and (B) methanol + 0.2% formic acid + 1 mM ammonium formate. In order to ensure robustness and reproducibility of the lipidomic analysis in AH, a panel of class-specific internal standards covering major lipid classes was spiked into each sample prior to extraction (SM 12:0, 300 pmol; Cer 12:0, 50 pmol; 15:0-18:1-d7-PC, 250 pmol; and 18:1-d7 Lyso PC, 250 pmol). These internal standards were used to correct for extraction efficiency, matrix effects, and instrument variability across the analytical sequence. Data normalization was performed using an internal standard–based approach, further adjusted for the extracted sample volume to ensure quantitative comparability of lipid profiles. Limits of detection (LOD) and quantification (LOQ) were estimated for representative lipid species based on signal-to-noise ratios of 3 and 10, respectively, corresponding to concentration ranges of approximately 1–5 nM (pmol/mL) for sphingolipids and 0.003–0.04 µM (pmol/µL) for phospholipids. Lipid species with signal intensities below the LOD were excluded from downstream analysis to minimize unreliable low-level measurements. Given that this study applied a previously validated method to a new biological matrix and did not develop a completely new assay, the analytical performance in AH was evaluated in a simple, fit-for-purpose way. The assessment focused on key matrix-specific factors such as extraction efficiency and sensibility, while keeping the original protocol and validation data.

### 2.5. Statistical Analysis

To assess differences in the mean concentration of individual biomarkers between groups, linear regression implemented in limma package in R environment [PMID: 25605792] was used to regress measures of metabolite concentrations (pg/mL for cytokines, pmol/mL for sphingolipids, pmol/μL for phospholipids) on case status (0/1) using patient age as covariate and on outcomes of interest (patient age, EZ width). Statistical significance was assessed using the *p*-value associated with the regression β coefficient (Pβ), with correction for multiple comparisons via the Benjamini–Hochberg false discovery rate (FDR) method.

## 3. Results

### 3.1. Population

A total of 39 patients with a clinical indication for cataract surgery were recruited for the study: 26 RP patients and 13 non-RP patients as controls. The RP cohort included 17 males and 9 females, with a mean age of 50.19 ± 13.90 years. RP was genetically confirmed in 20 out of 26 cases (76.9%). Among these, 10 had autosomal dominant inheritance, and 10 had autosomal recessive inheritance. For the remaining six patients, genetic testing is currently underway (Table 1). The control group included non-RP patients, 10 males and 3 females, with a mean age of 56.46 ± 7.76 years. All RP patients in the study cohort presented with posterior subcapsular cataract (PSC), the most frequent lens opacity in RP, with a reported prevalence of 41–53% [29,30]. In older RP patients, PSC was frequently accompanied by coexisting nuclear opacity. In contrast, control subjects exhibited age-related cataract, predominantly characterized by nuclear and/or cortical opacities, with no PSC component, in keeping with their otherwise healthy ocular status and the absence of conditions known to predispose to PSC formation (e.g., chronic intraocular inflammation, steroid use, high myopia, or diabetes).

### 3.2. Pro- and Anti-Inflammatory Cytokines Expression

The AH levels of various cytokines were analyzed in RP patients and control groups. The pro-inflammatory cytokine IL-6 levels were significantly elevated in RP patients compared to controls (median [interquartile range]; 4.31 [2.46–5.09] vs. 1.66 [0.86–2.15] pg/mL; logFC = −2.39) (Table 2). Vascular endothelial growth factor (VEGF) was found to be significantly lower in RP patients compared to the control group (17.34 [7.76–23.83] vs. 60.30 [45.89–81.25] pg/mL; logFC = 37.68) (Table 2). No statistically significant differences were observed for the other cytokines analyzed between RP patients and the control group.

### 3.3. Lipidomic Profile

Lipidomic analysis demonstrated a significant increase in medium- and long-chain SMs in RP patients compared to healthy controls. Additionally, very-long-chain unsaturated PCs were significantly elevated in RP patients. Although not statistically significant, Cer species carrying eighteen-carbons acyl chain (C18:0 Cer) also showed elevated levels in RP patients compared to controls (Table 2).

### 3.4. Correlation Between Biomarkers and Age in RP Group

**Figure 1:**
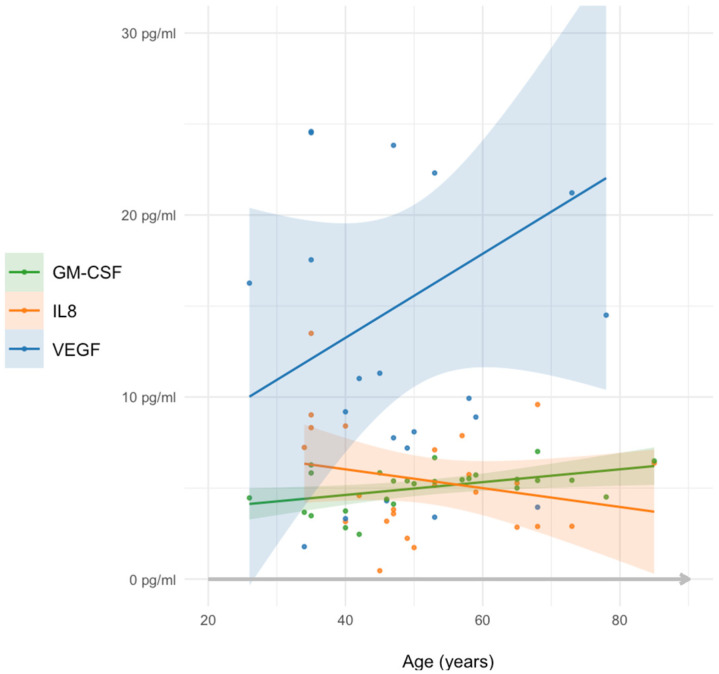
Correlation between biomarker levels and age in RP patients. VEGF (β = 0.267, R^2^ = 0.188) and GM-CSF (β = 0.035, R^2^ = 0.212) showed significant positive correlations with age, whereas IL-8 demonstrated a mild non-significant negative trend (β = −0.052, R^2^ = 0.053).

Biomarkers expression in the RP group was correlated with age. Although data dispersion was observed, as commonly occurs in clinical studies involving human ocular fluids, particularly in rare diseases, VEGF (*p* = 0.01) and GM-CSF (*p* = 0.02) levels exhibited a significantly positive correlation with increasing age, indicating an increase with advancing age. Conversely, IL-8 (*p* = 0.07) levels showed a decreasing trend with age, although this correlation did not reach statistical significance (Figure 1).

### 3.5. Correlation Between Biomarkers and EZ Width in RP Group

**Figure 2:**
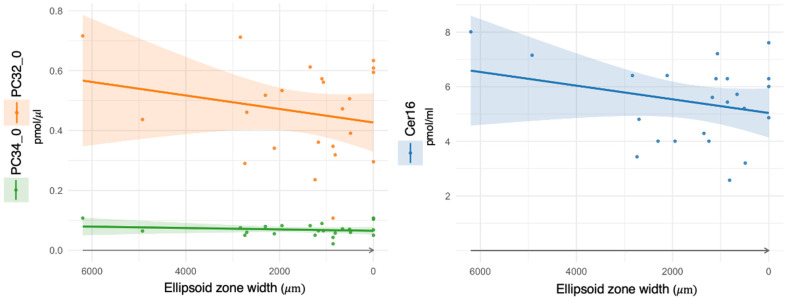
Correlation between biomarker levels and ellipsoid zone (EZ) width in RP patients. The *x*-axis representing EZ width is displayed in decreasing order to reflect the progressive reduction of this parameter with disease progression. Significant positive correlations were observed between EZ width and PC32:0 (β = 0.000051, R^2^ = 0.238), PC34:0 (β = 0.0000075, R^2^ = 0.188), and Cer16:0 (β = 0.00047, R^2^ = 0.227). Accordingly, positive correlations therefore indicate that higher biomarker levels are associated with more preserved retinal structure.

In RP patients, we further examined the relationship between biomarkers levels and the EZ width. A significant positive correlation was observed between EZ width and the pro-inflammatory C16:0 Cer (*p* = 0.03), as well as PC 32:0 (*p* = 0.02) and PC 34:0 (*p* = 0.04), indicating higher levels of these lipids in patients with a more preserved retinal architecture (Figure 2).

## 4. Discussion

Our study aimed to evaluate the concentration of key inflammatory proteins in the AH of patients affected by RP and, for the first time, to explore the lipid profile through mass spectrometry analysis. The link between oxidative stress, inflammation, and disease progression in retinal dystrophies is supported by an increasing body of scientific evidence [4,12,43].

In terms of proteins, our study confirmed findings from the literature: IL-6, a cytokine produced at sites of inflammation that plays a pro-inflammatory role both in the acute phase and by promoting the chronic inflammatory response, was significantly increased in RP patients compared to the control group. In RP, the disease trigger is the genetic variant causing the primary degeneration of rods. Therefore, damage-associated molecular patterns (DAMPs) are released, leading to microglial activation [44], which can affect cone survival. Lu et al. [14], Yoshida et al. [45], and De la Cámara et al. [46] previously reported significant differences in IL-6 levels in the AH of RP patients compared to controls, suggesting sustained chronic inflammation in the eyes of affected individuals. Pre-clinical data also corroborate this finding: increased levels of IL-6 have been observed in the retinas of rd10 mice, a commonly used RP model [45]. Additionally, studies have shown that treatments targeting IL-6 pathways can mitigate retinal damage [47].

Another notable difference in the cytokine group was the reduction in VEGF concentration in RP patients compared to controls. VEGF is a polypeptide secreted by tissues in response to hypoxia and plays a major role in vascular development and homeostasis regulation. It binds to receptors on vascular endothelial cells, promoting cell survival, proliferation, permeability, and migration. Our data confirm findings by [48,49]. A common pathway may explain this finding: in RP, the genetic mutation acts as the disease trigger, leading to the primary degeneration of rod cells. This event causes, on one hand, a state of hyperoxia, which is responsible for the decreased stabilization of HIF-1α, a key regulator of VEGF expression, and, consequently, reduced VEGF production [50]. On the other hand, it leads to the secondary degeneration of RPE cells, which lose their ability to support retinal function and contribute to reduced VEGF levels [51].

To the best of our knowledge, lipidomic analysis was performed for the first time on the AH of RP patients. Our results indicate that the most significant variations in lipid composition were found in PCs, SMs, and C18:0 Cer species. Lipid signaling is increasingly recognized as playing a role in the pathophysiological mechanisms of retinal disorders, including inflammation [52], cell death [53], and retinal damage [54]. The retina is highly enriched in polyunsaturated fatty acids, which undergo elongation to produce very long-chain omega-3 and omega-6 polyunsaturated fatty acids (VLC-PUFAs), primarily incorporated into PC lipids [55]. In the RPE, these lipid species downregulate SASP (senescence-associated secretory phenotype) and low-grade inflammation, thus maintaining photoreceptor integrity and function, as recently reviewed [56]. In a mouse model of bacterial endophthalmitis, Ahmad et al. observed a significant reduction in retinal PC levels 48 h post-infection [57]. Our analysis shows greater abundance of very-long-chain unsaturated PCs (PC 32:1, 34:1, 36:1, 38:2) in the AH of RP patients compared to healthy controls. These apparent mismatches may be explained by the weakening of the retinal barrier that loses components further released and accumulated in the patient AH. Similarly, the more permeable blood-retina barrier in diabetic patients promotes the transfer of glucose and phospholipids into the vitreous humor [58]. Beyond membrane damage, abnormal PC and SM levels have been linked to inflammatory conditions, though the ocular and retina-specific disorders are less explored. Bioactive lysophospholipids and sphingolipids potentially implicated in inflammation signaling have been found altered in proliferative diabetic retinopathy versus normal vitreous [22,23]. In an in vitro model of human epithelial corneal cell line mimicking dry eye chronic inflammatory condition, sphingolipids, glycerolipids, and glycerophospholipids display marked alterations [59]. Lipidomic analysis of AH from endotoxin-induced uveitis (EIU) rats during the acute inflammation stage showed a remarkable increase in total phospholipids (PC, PE, PS, PI, PG) as well as in total sphingolipids (ceramides and SM) in the EIU compared to the control group [60]. Indeed, PC and SM involvement in modulating inflammation processes has been widely described in intestinal-related disorders. A mouse model of ulcerative colitis showed increased PCs expression [61], which correlated with elevated IL-6 and TNF-α in Crohn’s disease-induced intestinal inflammation [62]. During the inflammatory process in inflammatory bowel disease, the PCs profile changes, impairing the mucus barrier. PCs are further metabolized by phospholipase A2 to release precursors of pro- and anti-inflammatory mediators [63].

Sphingolipids are a subclass of cellular lipids actively involved in cellular signaling, function regulation, and structural support [64]. Dysregulation of sphingolipids contributes to the development of various retinal diseases [65]. We observed a significant increase in medium- and long-chain SMs, likely due to altered retinal membrane structure in RP patients, with a consistent positive correlation with inflammation. In an in vivo model of brain ischemic stroke, the acute phase of neurodegeneration and inflammation was characterized by an elevation in myelin-associated lipids, including SMs [66]. In the mucosal lipid profile of colon biopsies from ulcerative colitis patients, Cer, SMs, and PCs profiles were the most dysregulated [67]. Additionally, recent reports show that the circulating SMs cycle is associated with the progression of RA, with the SM-(d18:0/18:2) species significantly increased in RA patients [68].

Among sphingolipids, Cer are bioactive mediators of apoptosis and serve as signaling molecules to activate inflammatory pathways [69], although not all Cer are equally harmful. In our study, we found that RP AH was enriched with C18:0 Cer, which has been implicated in inflammation. Elevated levels of C18:0 Cer and SMs species were strongly correlated with motor defects, neuroinflammation, and the expression of IL-6, IL-1β, and TNF-α [70]. Serum levels of C18:0 Cer were found elevated in obese individuals, characterized by chronic low-grade systemic inflammation with increased production of TGF-β1, IL-6, and TNF-α [71]. Furthermore, in the lower airway, cystic fibrosis patients show C18:0, C16:0, and C20:0 Cer accumulation, which has been linked to neutrophilic inflammation [72].

When considering both protein and lipid mediators in relation to age in affected individuals, we found a statistically significant positive correlation for VEGF and GM-CSF, while IL-8 decreases with age. Since RP is a progressive disease, aging is associated with disease progression, leading to a more chronic inflammatory response in the retina. In this context, microglial activation may increase GM-CSF levels to help mediate repair, contributing to higher levels of GM-CSF in older RP patients [73]. In contrast, IL-8 levels might decrease in the retina with aging because the recruitment of neutrophils becomes less essential in the chronic inflammation phase of retinal degeneration. As RP patients age, the retinal immune response may become more dominated by microglia rather than by neutrophil-driven responses [74].

Even though VEGF is statistically significantly reduced in RP patients compared to a control group, as previously described in this work, among RP patients, we observed an increase in VEGF level with age: this increase is likely secondary to chronic inflammation and oxidative stress, which are characteristic features of RP.

Among all the bioactive mediators, we observed a correlation between EZ width and both C16:0 Cer and PCs. Though C16:0 Cer is mainly involved in pro-inflammatory contexts, our analysis shows a slight positive correlation with EZ band width and thus with EZ structural integrity. In the early stages of retinal degeneration, as in younger patients, the EZ structure is almost preserved but is likely subjected to the worst inflammatory conditions driving disease progression and retinal damage. A few PCs species share the same trend, further supporting inflammation and the ensuing retinal alterations. Moreover, C16:0 Cer has been shown to activate the p53-mediated apoptotic pathway [75], which significantly affects photoreceptor viability in younger patients with preserved EZ. Indeed, previous studies in mouse models of RP have demonstrated that inhibition of de novo Cer synthesis using Myriocin has proven efficacious in slowing retinal degeneration [76,77].

## 5. Conclusions

In conclusion, our study demonstrates that patients with RP have higher levels of inflammatory molecules in the AH, confirming elevated levels of pro-inflammatory cytokines and adding for the first time new data about the lipidomic profile.

The main limitations of our study are the relatively small cohort and the genetic heterogeneity of our RP cohort, which encompasses several causative genes with potentially different disease mechanisms and progression patterns. The limited number of patients carrying each individual variant did not allow a robust genotype-stratified analysis. Nevertheless, the inflammatory and oxidative pathways downstream of photoreceptor degeneration are largely shared across genetic subtypes, supporting the biological consistency of our findings. Future studies on larger, genetically homogeneous cohorts will be required to determine whether specific genotypes are associated with distinctive AH proteomic and lipidomic signatures.

Furthermore, AH is a dynamically renewing fluid, and in our study, AH sampling was limited to the time of cataract surgery; therefore, it may not fully reflect the long-term inflammatory and metabolic status of the eye and precludes assessment of the temporal dynamics between biomarker changes and disease progression.

Due to the cross-sectional design and the limited sample size, this might be considered an exploratory study deserving further investigation with larger cohorts to validate these molecules as potential biomarkers.

## Figures and Tables

**Table 1:** Demographic and genetic characteristics of RP patients. Legend: HET, heterozygous; HOM, homozygous; ex, exon.

ID	Age at Baeline	Gene	RefSeq	Exon/Intron	Nucleotide Change I	Nucleotide Change II	Allelic State	References
RP1	42	*RHO*	NM_000539	ex4	c.403C>T	-	HET	[31]
RP2	40	*PRPF31*	NM_015629	ex2–8	del ex2–8	-	HET	NEW
RP3	35	*PRPF31*	NM_015629	ex7	c.551_552insCCGGAGCT	-	HET	NEW
RP4	35	*EYS*	NM_001142800	ex43; ex16–19	c.9299_9302del	del ex16–19	HET	[32]
RP5	26	*RDH12*	NM_152443	ex4	c.146C>T	c.146C>T	HOM	[33]
RP6	40	ongoing	-	-	-	-	-	-
RP7	34	*SNRNP200*	NM_014014	-	-	-	HET	-
RP8	35	*USH2A*	NM_206933	ex27	c.5418_5424del	c.5418_5424del	HOM	[34]
RP9	37	*PRPF31*	NM_015629	ex10	c.992G>A	-	HET	[35]
RP10	78	ongoing	-	-	-	-	-	-
RP11	45	*USH2A*	NM_206933	-	-	-	HET	-
RP12	47	*PRPF3*	NM_004698	ex11	c.1477C>T	-	HET	[36]
RP13	50	*EYS*	NM_001142800	-	-	-	HET	-
RP14	53	*USH2A*	NM_206933	ex69; ex2	c.14977_14978del	c.269A>G	HET	[34,37]
RP15	68	*RHO*	NM_000539	ex5	c.1033G>C	-	HET	[38]
RP16	49	ongoing	-	-	-	-	-	-
RP17	65	*USH2A*	NM_206933	ex7; ex63	c.1256G>T	c.13335_13347delinsCTTG	HET	[39,40]
RP18	65	ongoing	-	-	-	-	-	-
RP19	68	ongoing	-	-	-	-	-	-
RP20	57	*PRPH2*	NM_000322	ex1	c.374C>T	-	HET	[41]
RP21	47	*USH2A*	NM_206933	-	-	-	HET	-
RP22	53	*USH2A*	NM_206933	ex61; ex63	c.11864G>A	c.12574C>T	HET	[34,42]
RP23	58	ongoing	-	-	-	-	-	-
RP24	59	*RP1*	NM_006269	ex4	c.2447dup	-	HET	NEW
RP25	46	*USH2A*	NM_206933	-	-	-	-	-
RP26	73	*RP1*	NM_006269	ex4	c.2684T>G	-	HET	NEW

**Table 2:** Integrated proteomic and lipidomic profile of aqueous humor in RP and control groups. The table reports the mean ± standard deviation (SD), mean difference between the RP and control groups (Diff. RP-Ctr) t-statistic (t-stat), *p*-value, and Benjamini–Hochberg false discovery rate (FDR) adjusted-*p*-value (adj-*p*-value) for cytokines and lipid species. Mean cytokine concentrations are expressed in pg/mL, while mean lipid concentrations are reported in pmol/μL t-stat was calculated for each comparison as part of the statistical analysis, and significance was determined using corresponding raw *p*-values and FDR-adjusted *p*-values.

Biomarkers	Mean ± SD	Mean ± SD	Diff. RP-Ctr	t-Stat	*p*-Value	Adj-*p*-Value
	RP	Controls				
VEGF	17.34 ± 14.63	60.30 ± 27.17	−37.68	5.01	0.00001	0.0007
IL6	4.31 ± 2.53	1.66 ± 1.33	2.39	−3.54	0.001	0.01
SM 18:1	52.52 ± 29.66	13.96 ± 12.19	36.59	−4.23	0.0001	0.003
SM 18	103.20 ± 57.37	28.81 ± 24.37	69.51	−4.16	0.0002	0.003
SM 24:1	30.67 ± 21.76	9.81 ± 10.11	19.66	−3.07	0.004	0.03
SM 16	206.59 ± 131.20	80.15 ± 81.05	117.09	−2.92	0.006	0.03
SM 24	32.28 ± 21.96	11.65 ± 10.92	19.03	−2.91	0.006	0.03
PC 34:1	3.50 ± 1.37	2.01 ± 0.99	1.39	−3.23	0.003	0.02
PC 36:1	0.68 ± 0.26	0.41 ± 0.17	0.24	−2.99	0.005	0.03
PC 38:2	0.13 ± 0.08	0.06 ± 0.06	0.08	−2.65	0.01	0.05

## Data Availability

All data generated or analyzed during this study are included in this article. Further inquiries can be directed to the corresponding author.
